# Comparative Transcriptomics Reveals Features and Possible Mechanisms of Glucose-Mediated Soil Fungistasis Relief in *Arthrobotrys oligospora*

**DOI:** 10.3389/fmicb.2019.03143

**Published:** 2020-01-23

**Authors:** Tong Liu, Ying Huang, Xiang-Xiang Chen, Xi Long, Yun-He Yang, Ming-Liang Zhu, Ming-He Mo, Ke-Qin Zhang

**Affiliations:** ^1^State Key Laboratory for Conservation and Utilization of Bio-Resources in Yunnan, Yunnan University, Kunming, China; ^2^Yunnan of China National Tobacco Corporation, Kunming, China; ^3^Biocontrol Engineering Research Center of Plant Disease and Pest, Yunnan University, Kunming, China; ^4^Biocontrol Engineering Research Center of Crop Disease and Pest, Yunnan University, Kunming, China; ^5^Key Laboratory of Microbial Diversity in Southwest China, Ministry of Education, Yunnan Institute of Microbiology, Yunnan University, Kunming, China

**Keywords:** *Arthrobotrys oligospora*, soil fungistasis, comparative transcriptomics, biological control, glucose

## Abstract

Soil-borne pest diseases result in large annual agricultural losses globally. Fungal bio-control agents are an alternative means of controlling pest diseases; however, soil fungistasis limits the effect of fungal agents. Nutrients can relieve soil fungistasis, but the mechanisms behind this process remain poorly understood. In this study, we determined and quantified the transcriptomes of *Arthrobotrys oligospora*, a nematode-trapping fungus, derived from samples of fresh conidia, germinated conidia, soil fungistatic conidia, and glucose-relieved conidia. The transcriptomes of fungistatic and glucose-relieved conidia were significantly different from those of the other two conidia samples. KEGG pathway analyses showed that those genes upregulated in fungistatic and glucose-relieved conidia were mainly involved in translation and substance metabolism, and the downregulated genes were mainly involved in MAPK pathway, autophagy, mitophagy, and endocytosis. As being different from the transcriptome of fungistatic conidia, upregulated genes in the transcriptome of glucose-relieved conidia are also related to replication and repair, spliceosome, oxidative phosphorylation, autophagy, and degradation pathway (lysosome, proteasome, and RNA degradation). And the upregulated genes resulted from comparison of glucose-relieved conidia and fungistatic conidia were enriched in metabolic pathways, cycle, DNA replication, and repair. The differentially splicing events in the transcriptome of glucose-relieved conidia are far more than that of other two transcriptomes, and genes regulated by differentially splicing were analyzed through KEGG pathway analysis. Furthermore, autophagy genes were proved to play important role in resisting soil fungistasis and glucose-mediated soil fungistasis relief. These data indicate that, in addition to being a carbon and energy source for conidia germination, glucose may also help to relieve soil fungistasis by activating many cellular processes, including autophagy, DNA replication and repair, RNA alternative splicing, and degradation pathways.

## Introduction

Soil-borne pest diseases are responsible for large agricultural losses. For example, plant–parasitic nematodes cause approximately $US 157 billion of global agricultural loss annually (Abad et al., 2008). Owing to the environmental toxicity of chemical pesticides, fungal bio-control agents were developed to control pest diseases in soil (Butt et al., 2001). However, soil is a complicated ecological environment, and soil fungistasis (mycostasis) strongly represses the germination and growth of fungal bio-control agents (Fang et al., 2011), rendering bio-control agents inefficient in soil. Soil fungistasis was identified in 1953 as a phenomenon that caused the inhibition of conidial germination and hyphal growth in soils (Dobbs and Hinson, 1953). Reaching a sufficient population density is a prerequisite for fungal agents to function effectively in soils. Soil fungistasis is prevalent in most soils (Lockwood, 1977; Ann, 1994; Fang et al., 2011), making the relief of soil fungistasis necessary for fungal agents to reach a sufficient population density.

Past decades studies argued that the most likely explanation for fungistasis is probably a combination of the nutrient-deficiency and fungistatic factor-mediated inhibition (Garbeva et al., 2011). Previous studies have also shown that soil supplementation with nutrients, including simple sugars, amino acids, corncobs, tapioca, and soybean powder, can relieve fungistasis (Lockwood, 1964, 1977). Nutrient combinations and complex materials such as plant residues are typically more effective than simple nutrients. Fungistasis relief by amino acids is greater in acidic soils, but the efficacy of glucose is unaffected by changes in soil pH (Emmatty and Green, 1967). Palmitic acid, octadecanoic acid, and citric acid can also promote the germination of endospores and chlamydospores (Papavizas and Kovacs, 1972; Lockwood, 1977). Based on these studies, bio-control agents have been developed by combining nutrients with fungal agents, and the spore germination rate of bio-control agents is improved in soil (Sun et al., 1997). However, the fungistasis relief effect of nutrients was shown to be temporary. For example, the spore germination rate of *Thielaviopsis basicola* was improved for the first 4 days following the addition of alfalfa hay in soil, but greater fungistasis was observed after the fifth day (Adams and Papavizas, 1969).

Although many compounds can relieve soil fungistasis, the underlying mechanisms remain poorly understood. It was speculated that fatty acids are broken down by acid-metabolizing microorganisms to simple compounds that have stimulatory activity on fungal germination (Papavizas and Kovacs, 1972). Some plant-pathogenic fungi can prevent accumulation of toxins in the cell to toxic concentrations through the function of ABC transporter which is an energy-driven (ATP or proton motive force) efflux pump (Schouten et al., 2008). Although Garbeva et al. (2011) indicated that energy-driven efflux pumps might help fungi to resist soil fungistasis, and the exogenous nutrients in soil would provide energy for efflux pumps, this speculation remains to be proved by further research. Except for these reports, there is little report about the mechanisms behind soil fungistasis relief by nutrients. Further researches are very necessary to elucidate the underlying mechanisms.

*Arthrobotrys oligospora* is a nematode-trapping fungus that is widespread in soils. It is used to control plant-parasitic nematodes, especially *Meloidogyne* spp. (Singh et al., 2012, 2013). *A. oligospora* conidial germination is repressed by soil fungistasis (Liu, 2019), making it a suitable model organism for researching the mechanisms behind soil fungistasis relief by nutrients. In recent years, omics techniques have been widely used to study scientific topics of interest in fungi. For example, proteomics was used to understand the molecular mechanisms of conidial germination (Oh et al., 2010; Deng et al., 2015). Transcriptomics was used to elucidate the divergent lifestyle features of the nematode endoparasitic fungus *Hirsutella minnesotensis* (Lai et al., 2014). In addition, comparative transcriptomics revealed the different strategies of *Trichoderma* mycoparasitism (Atanasova et al., 2013). These studies suggest that omics techniques can be used to elucidate the mechanisms behind soil fungistasis relief by nutrients. In this study, the transcriptomic features and possible mechanisms of glucose-induced soil fungistasis relief were revealed based on comparative transcriptomics of *A. oligospora* conidia.

## Materials and Methods

### Preparation of Conidia Samples for Transcriptomic Analysis

*Arthrobotrys oligospora* strain ATCC 24927 was cultured on corn meal agar (CMA) plates at 28°C for sporulation. The conidia were collected as per previously described method (Liu et al., 2018). Four conidia samples were prepared, namely fresh, germinated, fungistatic, and glucose-relieved conidia. Conidia harvested from fresh cultures were deemed to be fresh. To prepare germinated conidia, fresh conidia were allowed to germinate on water agar plates at 28°C for 24 h and then harvested. In order to prepare fungistatic conidia, several kilograms of surface soils (20 cm surface layer) were gathered from Huainan, Yunnan, China. Soil and deionized water were added into a breaker at a mass ratio of 2.5:1, and were mixed well to produce about 300 ml soil suspension. In order to prepare fungistatic conidia, approximately 300 mg of fresh conidia were suspended with 3 ml of sterilized water, and the conidia suspension was transferred into a dialysis bag (300 KD; Spectrum, United States) pretreated as per previously reported method (Liu, 2019). The dialysis bag was then placed into the 300 ml soil suspension for 24 h, with agitation of the soil suspension by a magnetic stirrer. The glucose-relieved conidia were prepared by adding 1 ml of 1% glucose (w/v) into a dialysis bag with the same conidia suspension, and were also placed into the 300 ml soil suspension. Totally, two fungistatic conidia samples and two glucose-relieved conidia samples were placed into the 300 ml soil suspension. The glucose concentration of soil suspension was assessed by using Roche blood glucose meter (Roche Ltd., Shanghai, China) (Supplementary Table S1). All four conidia samples had two duplicates. The fresh conidia sample, germinated conidia sample, fungistatic conidia sample, and glucose-relieved conidia sample were named AO-Ck, AO-G24, AO-So, and AO-Re, respectively.

### Transcriptome Sequencing

For total RNA extraction, approximately 300 mg of conidia sample was broken up by grinding with liquid nitrogen, and was treated following the protocol of the Ambion PureLink^®^ RNA Mini Kit (Invitrogen, United States). The total RNA was sent to the Beijing Genomics Institute (BGI, China) for transcriptome sequencing using an Illumina HiSeq 4000 sequencer. After sequencing, we obtained the raw reads. Firstly, we filtered the low-quality reads [a read in which the percentage of low-quality bases (<10) was >20%], adaptor-polluted reads, and reads with a high content of unknown bases (>5%) to obtain clean reads. The clean reads were stored in the FASTQ (Cock et al., 2010) format, and the clean reads data were deposited in National Omics Data Encyclopedia^1^ (project ID, OEP000440).

### Genome Mapping

After clean-read filtering, we used HISAT (version: 0.1.6-beta; parameter: –phred64–sensitive–no-discordant–no-mixed-I1-X1000) (Kim et al., 2015) to perform genome mapping (the genome of *A. oligospora* strain ATCC 24927 in GenBank was used as a reference genome).

### Novel Transcript Prediction

A novel transcript can be both a new isoform of a previously known gene or a transcript without any known features. We used StringTie (version: v1.0.4; parameters: -f0.3-j3-c5-g100-s10000-p8) (Pertea et al., 2015) to reconstruct the transcripts, and CuffCompare (version: v2.2.1; parameters: -p12) to identify novel transcripts by comparing reconstructed transcripts to reference annotations. We then used CPC (version: v0.9-r2; parameters: default) (Kong et al., 2007) to predict the coding potential of novel transcripts, and merged the novel coding transcripts with the reference transcripts to obtain a complete reference, which was used as the basis for downstream analysis.

### Detection of Differentially Spliced Genes

We used rMATS (Shen et al., 2014) to detect differentially spliced genes (DSGs) and calculate the isoform ratio of a gene between two conidia samples. Five alternative splicing events were detected: skipped exon (SE), alternative 5′-splicing site (A5SS), alternative 3′-splicing site (A3SS), mutually exclusive exons (MXE), and retained intron (RI). The statistical model of rMATS (version: v3.0.9; parameters: -analysis U -t paired -a8) calculates the *P*-value and false discovery rate (FDR) of the difference in the isoform ratio. Genes with FDR ≤ 0.05 are defined as DSG.

### Gene Expression Analysis and Detection of Differentially Expressed Genes

We mapped the clean reads to the complete reference using Bowtie2^2^ (Langmead and Salzberg, 2012), and then calculated gene expression levels using RSEM^3^ (Li and Dewey, 2011). The mapped gene was defined as expressed gene. We also calculated the overlap of expressed genes between samples and showed the result with a Venn diagram. Using the gene expression level in fresh conidia as a control, we detected differentially expressed genes (DEGs) in other conidia samples with PossionDis (Audic and Claverie, 1997). Genes with a fold change of expression ≥3.00 and FDR ≤ 0.001 were considered differentially expressed.

### KEGG Pathway/Pathway Enrichment Analysis

We performed the Kyoto Encyclopedia of Genes and Genomes (KEGG) orthology-based annotation of genes based on previously reported methods (Liu et al., 2018). Then, we mapped the DEGs to the KEGG pathway database and KEGG BRITE database by using the KEGG mapper tool^4^. With the KEGG annotation result, we also performed pathway functional enrichment using phyper, a function of R program. The expressed gene data, and DEGs in AO-G24 and AO-Re, were used for pathway enrichment analysis. The *p*-value was calculated according the method on web of Wikipedia^5^. Then the FDR correction was performed, and pathway with a FDR ≤ 0.01 was considered as significant enrichment.

### Gene Mutation and Conidia Germination Rate Assays

In order to knock out genes *AOL_s00076g234* and *AOL_s00076g70* encoding autophagy proteins ATG1 and ATG5, respectively, the gene disruption vectors were constructed using backbone plasmid pRS426 and hygromycin cassette (hph) source vector pCSN44 according to the described method (Liu et al., 2018). The primers used for PCR amplification are listed in Supplementary Table S2. The gene disruption vectors were transformed into *A. oligospora* by a protoplast-based protocol (Tunlid et al., 1999; Leng et al., 2008), and hygromycin-resistant transformants were selected on PDASS containing 200 μg/ml hygromycin B (Leng et al., 2008), and were further confirmed by PCR. The conidia of wild-type and mutant strains were harvested, and then the conidia germination rate in water agar medium or soil suspension was tested by using microscope. In every test, about 200 conidia were counted and 3 replicates were performed. Conidium was considered as germinated when the length of its germ tube was of the same size or longer than its diameter.

## Results

### Test of Soil Fungistasis and Preparation of Conidia Samples

**FIGURE 1:**
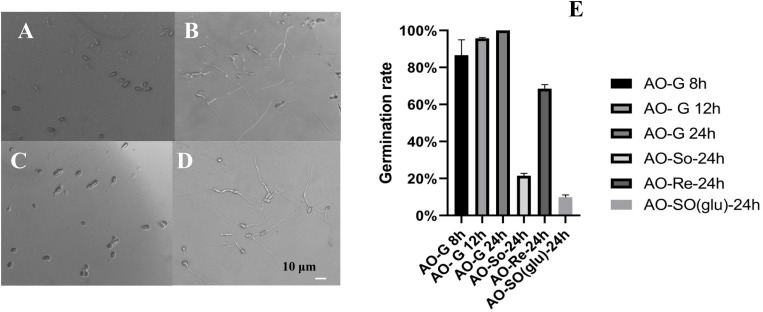
Microscopic observation of conidial germination. **(A)** Fresh conidia; **(B)** germinated conidia; **(C)** soil fungistatic conidia; **(D)** glucose-relieved conidia. **(E)** Statistics of conidial germination rate. AO-G, germinated conidia; AO-So, soil fungistatic conidia; AO-Re, glucose-relieved conidia; AO-SO(glu), fungistatic conidia in soil suspension (soil/water = 2.5/1) added with 3 ml of 1% glucose.

For the preparation of fungistatic conidia sample, different soil suspensions with different mass ratios of soil and water were used to test the germination rate of *A. oligospora* conidia (data not shown). We found that the soil suspension with a mass ratio of 2.5/1 is suitable for preparing fungistatic conidia and glucose-relieved conidia. As shown in Figure 1, the conidia germination of all four conidia samples was observed by microscope (Figures 1A–D). More than 85% conidia germinated at 8 h on water agar plate, and the germination rates at 12 and 24 h were about 95 and 100%, respectively (Figure 1E). The germination rate of fungistatic conidia at 24 h was about 20%, and the germination rate of glucose-relieved conidia at 24 h was about 70%. These results showed the fungistatic role of soil suspension on conidia germination, and glucose-mediated relief of soil fungistasis.

For preparation of glucose-relieved conidia, 1 ml of 1% glucose (w/v) was added into 3 ml conidia suspension. The initial theoretical glucose concentration was 13.9 mmol/L, and the tested glucose concentration was 14.8 mmol/L (Supplementary Table S1). The tested glucose concentration was slightly higher than the theoretical glucose concentration; this can be owed to the measurement error of glucose meter or interference of conidia suspension. The glucose would diffuse from the dialysis bag into soil suspension in a short time, and total 2 ml of 1% glucose (w/v) from two dialysis bag containing glucose-relieved conidia could diffuse into soil suspension. The theoretical maximum glucose concentration in soil suspension was about 0.35 mmol/L. Because of the glucose depletion by glucose-relieved conidia and soil microorganism in soil suspension, the glucose concentration in the dialysis bag containing fungistatic conidia was far <0.35 mmol/L. And it was confirmed that direct addition of 2 ml of 1% glucose (w/v) into soil suspension could not relieve the soil fungistasis [AO-SO(glu) in Figure 1E]. This meant that the early exposure to 13.9 mmol/L glucose mediated the relief of soil fungistasis, and the diffused glucose could not activate the conidia germination of fungistatic conidia.

### Overview of the Transcriptomes

**FIGURE 2:**
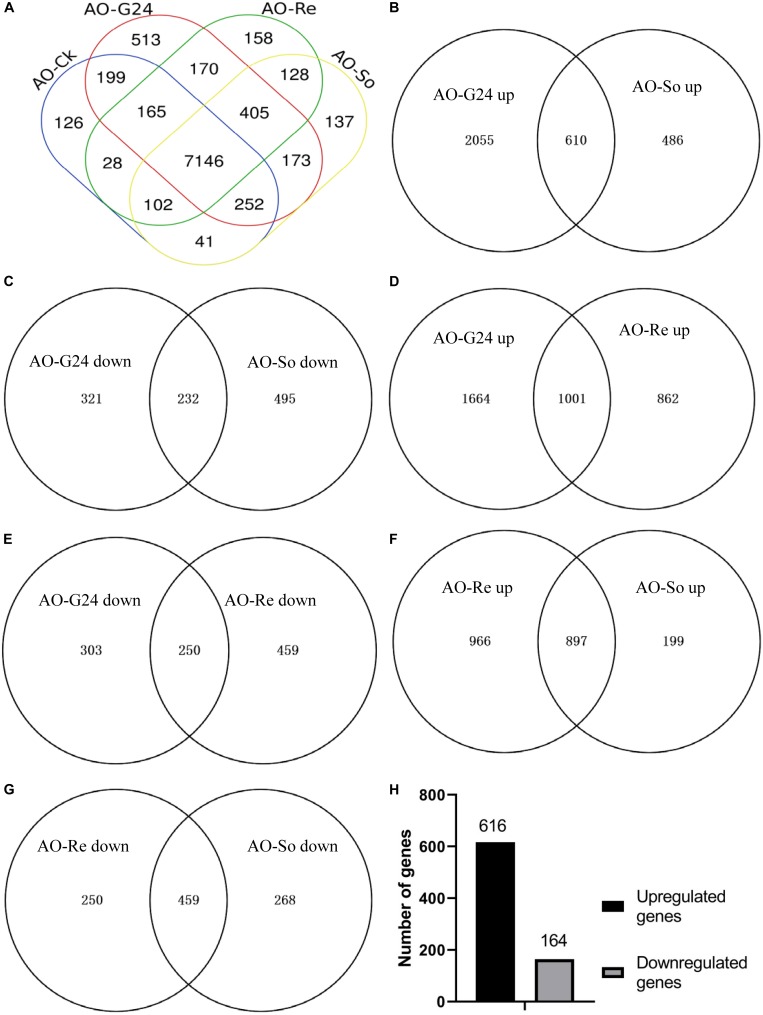
Venn diagram analysis of DEGs. **(A)** Comparison of the expressed genes in the four samples. **(B)** Comparison of the upregulated DEGs between AO-So and AO-G24. **(C)** Comparison of the downregulated DEGs between AO-So and AO-G24. **(D)** Comparison of the upregulated DEGs between AO-Re and AO-G24. **(E)** Comparison of the downregulated DEGs between AO-Re and AO-G24. **(F)** Comparison of the upregulated DEGs between AO-Re and AO-So. **(G)** Comparison of the downregulated DEGs between AO-Re and AO-So. **(H)** Statistics of differentially regulated genes in AO-Re (the transcriptome of AO-So was used as control). AO-G24, germinated conidia; AO-So, fungistatic conidia; AO-Re, glucose-relieved conidia; up, upregulated genes; down, downregulated genes.

Total RNA was extracted from the four types of conidia samples (Supplementary Table S3), used for library construction, and sequenced using the Illumina Hiseq platform. On average, approximately 4.46 Gb sequencing data were generated for each sample. The post-filtering read quality metrics are shown in Supplementary Table S4. The distribution of the base content and quality is shown in Supplementary Figure S1. The clean reads were mapped to the *A. oligospora* reference genome. On average, 80.80% reads were mapped, and the uniformity of the mapping result for each sample suggests that the samples are comparable (Supplementary Table S5). When the clean reads were mapped to the complete reference (Table 1), 55.12–70.01% clean reads were mapped. More than 6600 novel transcripts were detected in each sample, and more than 8000 known genes were expressed in each sample. There were 7146 common genes expressed in all samples (Figure 2A).

**TABLE 1:** Summary of gene expression.

Samples	Total clean reads	Total mapping ratio (%)	Uniquely mapping ratio (%)	Total gene number	Known gene number	Novel gene number	Total transcript number	Known transcript number	Novel transcript number
AO-Ck	30267082	70.01	28.13	8059	8043	16	14,698	7995	6703
AO-G24	29541820	69.82	28.82	9023	8996	27	16,765	9450	7315
AO-Re	30213230	55.12	26.37	8302	8258	44	12,889	6208	6681
AO-So	29406102	67.33	30.45	8384	8354	30	14,454	7578	6876

Using the gene expression levels in fresh conidia as a control, genes in other samples that had a fold change ≥ 3 and FDR ≤ 0.001 were considered as DEGs. There were 2665 upregulated genes and 553 downregulated genes in germinated conidia, but only 1096 upregulated genes and 727 downregulated genes in fungistatic conidia (Figures 2B,C). Germinated and fungistatic conidia shared only 610 upregulated genes and 232 downregulated genes. Changes in the transcriptome of the glucose-relieved conidia were also observed, specifically 1863 upregulated genes and 709 downregulated genes (Figures 2D,E). Germinated and glucose-relieved conidia shared 1001 upregulated genes and 250 downregulated genes. DEGs in fungistatic and glucose-relieved conidia were also compared using a Venn diagram. There were 897 common upregulated genes (Figure 2F) and 459 common downregulated genes (Figure 2G); however, 966 upregulated genes and 250 downregulated genes in glucose-relieved conidia were not differentially expressed in fungistatic conidia. Moreover, compared with fungistatic conidia, 616 genes were upregulated and 163 genes were downregulated in glucose-relieved conidia (Figure 2H). The DEGs lists are provided in Supplementary Data Sheet S2.

These results suggest that the transcriptome of fungistatic conidia differs considerably from that of germinated conidia, and the transcriptome of conidia under soil fungistasis is dramatically changed by the presence of glucose.

### Characterization of the Fungistatic Conidia Transcriptome and Glucose-Relieved Conidia Transcriptome

In order to characterize the difference in transcriptomes of germinated and fungistatic conidia, and the difference in transcriptomes between germinated and glucose-relieved conidia, sets of unique DEGs in fungistatic and glucose-relieved conidia were analyzed through KEGG pathway analysis (Table 2).

**TABLE 2:** Pathway analysis of differentially expressed genes in transcriptomes of fungistatic and glucose-relieved conidia.

KEGG pathway at second level	KEGG pathway at third level	AO-So		AO-Re	
		Up	Down	Up	Down
Global and overview maps	Metabolic pathways	71	69	106	63
	Biosynthesis of secondary metabolites	26	23	35	24
	Biosynthesis of antibiotics	22	18	31	16
	Microbial metabolism in diverse environments	11	20	25	14
	Carbon metabolism	4	9	7	6
	Biosynthesis of amino acids	15	1	16	6
Translation	Ribosome	51	4	51	5
	Ribosome biogenesis in eukaryotes	15	1	13	1
	RNA transport	8	4	9	4
	Aminoacyl-tRNA biosynthesis	3	1	6	2
	mRNA surveillance pathway	2	2	7	0
Transcription	RNA polymerase	3	3	4	1
	Spliceosome	6	4	14	2
Energy metabolism	Oxidative phosphorylation	1	1	7	3
Carbohydrate metabolism	Fructose and mannose metabolism	4	4	6	3
	Starch and sucrose metabolism	7	3	8	3
Lipid metabolism	Glycerophospholipid metabolism	3	4	5	5
Nucleotide metabolism	Purine metabolism	8	3	9	3
Amino acid metabolism	Cysteine and methionine metabolism	7	1	7	3
	Glycine, serine, and threonine metabolism	4	5	6	3
Folding, sorting, and degradation	Protein processing in endoplasmic reticulum	4	3	9	5
	RNA degradation	2	3	8	3
	Protein export	2	0	8	0
	Proteasome	0	2	5	0
Replication and repair	DNA replication	2	0	5	1
	Nucleotide excision repair	4	2	6	3
	Base excision repair	1	1	4	1
	Mismatch repair	2	0	4	0
Signal transduction	MAPK signaling pathway – yeast	6	13	8	14
Transport and catabolism	Peroxisome	8	3	6	4
	Autophagy – yeast	3	10	4	6
	Endocytosis	2	9	7	6
	Mitophagy – yeast	4	5	3	5
	Phagosome	4	1	5	3
Cell growth and death	Cell cycle – yeast	8	4	9	8
	Meiosis – yeast	7	7	7	9
Environmental adaptation	Thermogenesis	4	2	10	5

The most obvious common feature revealed by KEGG pathway analysis is that 79 and 86 upregulated genes in transcriptomes of fungistatic and glucose-relieved conidia, respectively, are related to translation. These genes are involved in ribosome, ribosome biogenesis, RNA transport, aminoacyl-tRNA biosynthesis, and mRNA surveillance pathway. Dozens of upregulated genes are involved in carbohydrate, lipid, nucleotide, and amino acid metabolism, and the gene numbers of these pathways in fungistatic transcriptome are very close to that of glucose-relieved conidia. For the downregulated genes, 13 and 14 downregulated genes in transcriptomes of fungistatic and glucose-relieved conidia, respectively, are involved in MAPK pathway. Fifteen and 11 downregulated genes in transcriptomes of fungistatic and glucose-relieved conidia, respectively, are related to autophagy and mitophagy.

Moreover, the most obvious different feature of two transcriptomes is that more genes related to spliceosome, oxidative phosphorylation, protein processing in endoplasmic reticulum, RNA degradation, protein export, and proteasome are upregulated in the transcriptome of glucose-relieved conidia, as well as 19 upregulated genes related to replication and repair.

### Characterization of the Transcriptome Change After the Addition of Glucose

The *A. oligospora* conidia germinated early on water agar medium and entered into the stage of hyphal growth at 24 h. And the preparation treatment of glucose-relieved conidia was similar to that of fungistatic conidia. So, the transcriptome of fungistatic conidia is a better control than that of germinated conidia for revealing the mechanisms of glucose-mediated soil fungistasis relief. Aside from common DEGs, 966 genes were upregulated (Figure 2F) and 250 genes were downregulated (Figure 2G) in the glucose-relieved conidia. Subsequent KEGG pathway analyses of these DEGs were performed (Table 3).

**TABLE 3:** Pathway analysis of differentially expressed genes in transcriptomes of glucose-relieved conidia.

KEGG pathway at second level	KEGG pathway at third level	Upregulated gene	Downregulated gene
Global and overview maps	Metabolic pathways	123	41
	Biosynthesis of secondary metabolites	33	16
	Biosynthesis of antibiotics	26	12
	Biosynthesis of amino acids	11	8
Translation	Ribosome	6	0
	RNA transport	5	1
	mRNA surveillance pathway	7	2
Transcription	Spliceosome	8	1
Energy metabolism	Oxidative phosphorylation	9	0
Lipid metabolism	Glycerophospholipid metabolism	3	3
Amino acid metabolism	Glycine, serine, and threonine metabolism	5	3
	Tyrosine metabolism	4	3
	Cysteine and methionine metabolism	4	3
	Tryptophan metabolism	4	2
Carbohydrate metabolism	Inositol phosphate metabolism	4	6
	Glyoxylate and dicarboxylate metabolism	4	2
	Pentose and glucuronate interconversions	8	0
Glycan biosynthesis and metabolism	*N*-Glycan biosynthesis	7	1
Folding, sorting, and degradation	Protein processing in endoplasmic reticulum	7	5
	Ubiquitin-mediated proteolysis	4	2
	Proteasome	6	0
	RNA degradation	8	1
Replication and repair	DNA replication	9	1
	Mismatch repair	9	0
	Nucleotide excision repair	11	0
	Homologous recombination	7	0
Signal transduction	MAPK signaling pathway – yeast	7	8
	mTOR signaling pathway	4	3
	Phosphatidylinositol signaling system	3	6
Transport and catabolism	Mitophagy – yeast	8	1
	Autophagy – yeast	7	1
	Lysosome	5	2
	Endocytosis	8	0
Cell growth and death	Meiosis – yeast	5	5
	Cell cycle – yeast	8	5

Compared to the result in Table 2, more remarkable feature is revealed in Table 3. Thirty-six upregulated genes are related to replication and repair; 28 upregulated genes are related to autophagy, mitophagy, lysosome, and endocytosis; and few downregulated genes are found in these pathways. Besides, a lot of upregulated genes were also involved in translation, spliceosome, oxidative phosphorylation, and degradation pathway (proteasome and RNA degradation).

### Glucose-Relieved Fungistasis Is Different From Normal Germination

**FIGURE 3:**
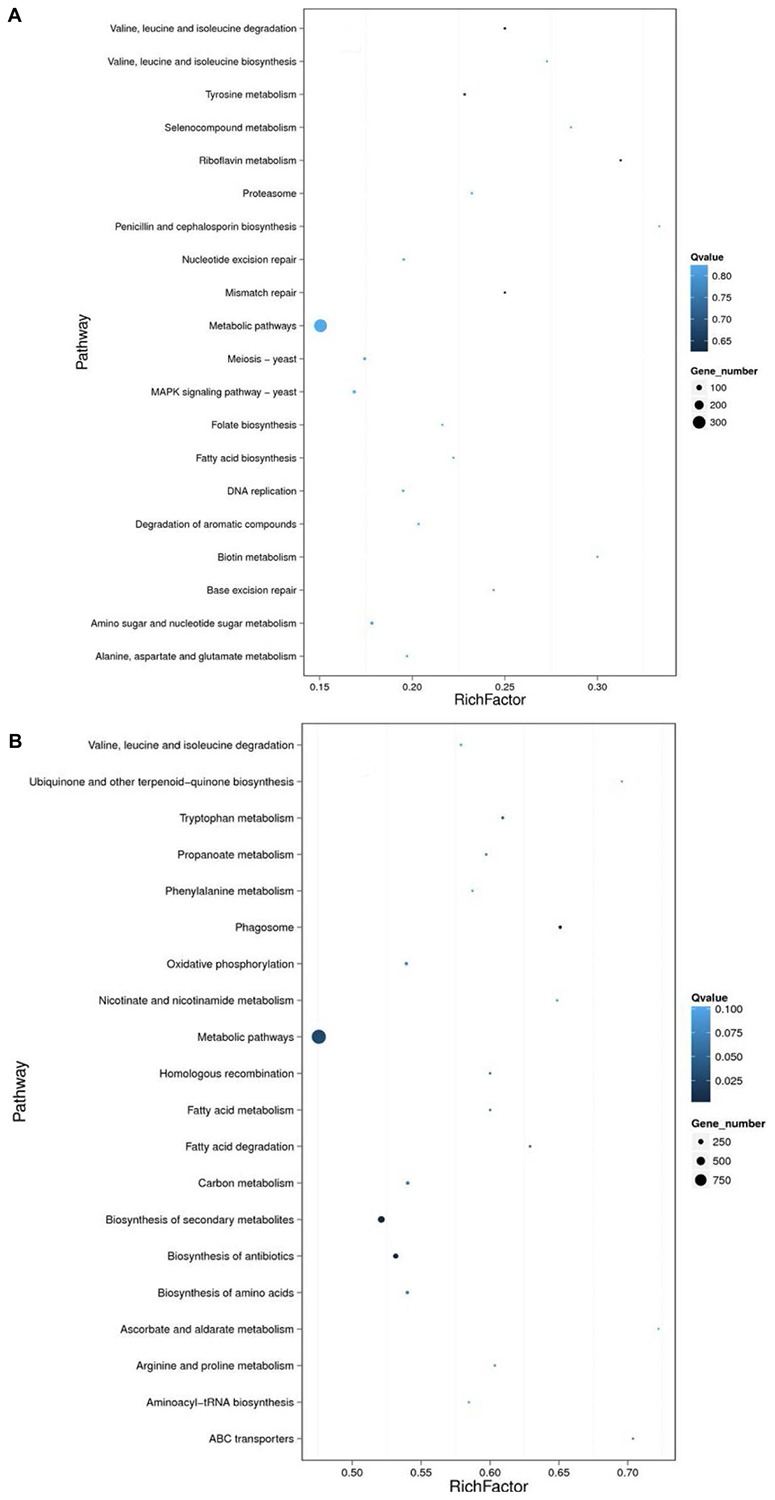
KEGG pathway enrichment analysis. The DEGs in comparison groups AO-Re/AO-So **(A)** and AO-G24/AO-Ck **(B)** were analyzed through KEGG pathway enrichment analysis. AO-Ck, fresh conidia; AO-G24, germinated conidia; AO-So, fungistatic conidia; AO-Re, glucose-relieved conidia.

Compared to the transcriptome of fresh conidia, there were 4284 DEGs (3361 upregulated genes and 923 downregulated genes) that had a fold change ≥ 2 and FDR ≤ 0.001. By contrast, using the transcriptome of fungistatic conidia as a control, there were 1457 DEGs (1152 upregulated and 305 downregulated genes) in glucose-relieved conidia that had a fold change ≥ 2 and FDR ≤ 0.001. And KEGG pathway enrichment analyses of these DEGs were performed (Figure 3).

For the DEGs in glucose-relieved conidia, the most enriched pathway was “metabolic pathways.” Besides, pathways related to cell cycle, DNA replication, and repair were also enriched, including “proteasome,” “meiosis,” “DNA replication,” “folate biosynthesis,” “nucleotide excision repair,” “mismatch repair,” and “base excision repair” (Figure 3A). Moreover, DEGs were also enriched in “MAPK signaling pathway.” These results were consistent with the results showed in Table 3. For the DEGs in germinated conidia, the most enriched pathway was “metabolic pathways.” Pathways related to amino acids metabolism, fatty acid metabolism, and secondary metabolism were also enriched (Figure 3B). Besides, DEGs were also enriched in pathways related to “phagosome,” “oxidative phosphorylation,” and “homologous recombination.” These results suggested that the enrichment pathways of glucose-relieved conidia are different from that of germinated conidia, and glucose-relieved fungistasis is different from normal germination.

### Analyses of Differentially Splicing Genes in Transcriptome of Glucose-Relieved Conidia

**FIGURE 4:**
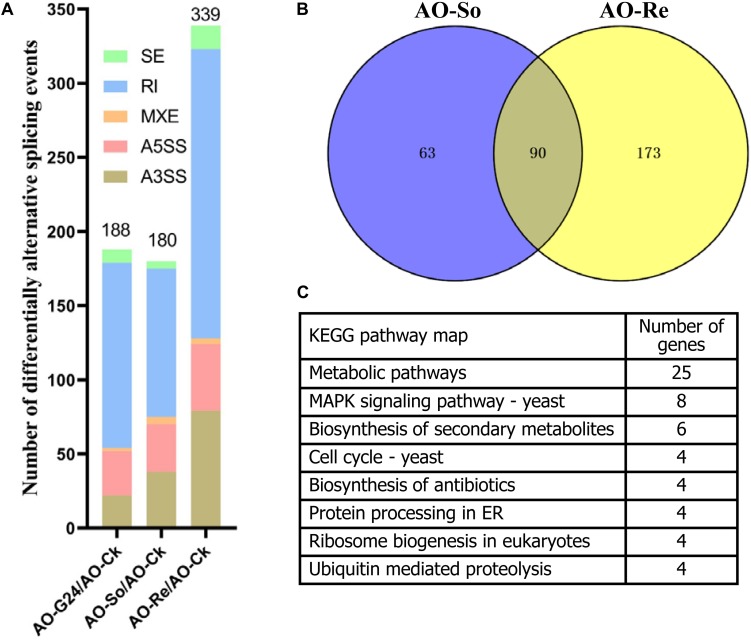
Analyses of differentially splicing events. **(A)** Statistics of differentially splicing events. **(B)** Comparison of genes regulated by differentially alternative splicing. **(C)** KEGG pathway analysis of genes regulated by differentially alternative splicing. AO-Ck, fresh conidia; AO-G24, germinated conidia; AO-So, fungistatic conidia; AO-Re, glucose-relieved conidia.

The transcriptome is also affected by the changes in abundance of transcriptional splicing isoforms. Changes in the splicing isoforms of a gene indicate a splicing-based gene expression regulatory mechanism. Fourteen genes related to the spliceosome were upregulated in glucose-relieved conidia, which suggests that gene expression regulation via alternative splicing in fungistatic conidia is affected by the addition of glucose. Five types of alternative splicing events were detected from transcriptome analysis. As shown in Figure 4, the number of differential splicing events increased markedly in glucose-relieved conidia (Figure 4A), further supporting that alternative splicing is affected by the addition of glucose. In the transcriptome of glucose-relieved conidia, 339 alternative splicing events are involved in transcription regulation of 263 genes (Figure 4B). Aside from common genes in the transcriptome of fungistatic conidia, 173 genes were analyzed through KEGG pathway analysis (Figure 4C). Eight, four, four, four, and four genes are involved in MAPK signaling pathway, cell cycle, protein processing in endoplasmic reticulum, ribosome biogenesis, and ubiquitin-mediated proteolysis, respectively. However, how alternative splicing influences conidial germination requires further study.

### Autophagy Functions in Conidia Resistance of Soil Fungistasis

**FIGURE 5:**
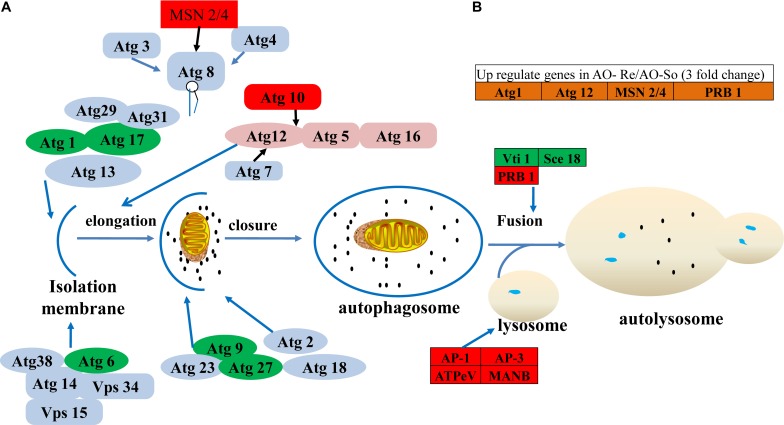
DEGs in the autophagy pathway. **(A)** Autophagy process in fungi. **(B)** Autophagy-related genes upregulated in glucose-relieved conidia compared with those in fungistatic conidia. Downregulated and upregulated genes are indicated with green and red background, respectively.

Ten genes related to the autophagy pathway were downregulated in fungistatic conidia compared with fresh conidia. Some of these autophagy pathway genes are directly involved in autophagy (Figure 5A). Genes *Atg1*, *Atg6*, *Atg9*, *Atg17*, and *Atg27*, as well as *Vti1* and *Sec18*, which are involved in the fusion of autophagosomes and lysosomes, were downregulated in fungistatic conidia. By contrast, seven autophagy pathway genes were upregulated in glucose-relieved conidia, including MSN genes which promote the expression of ATG8 (Vlahakis et al., 2017). Moreover, four of these autophagy-related genes exhibited more than threefold increased expression in glucose-relieved conidia compared with that in fungistatic conidia (Figure 5B), although two of these four genes, namely *Atg1* and *Atg12*, were not differentially expressed between fresh conidia and glucose-relieved conidia.

**FIGURE 6:**
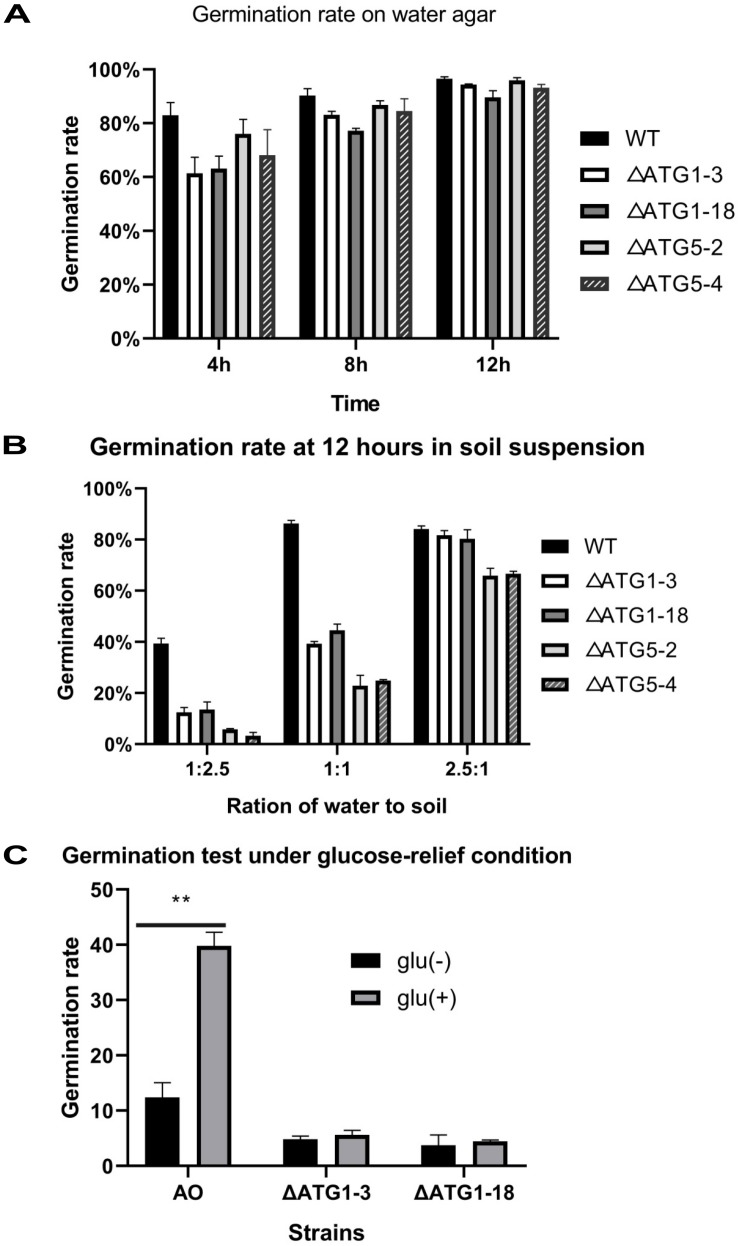
Conidia germination rate of the wild-type and mutant strains. Germination rates were determined on water agar medium **(A)** and in soil suspension **(B)**. Δ*ATG*1-3 and Δ*ATG*1-18: two *Atg1* knock-out mutant strains. Δ*ATG*5-2 and Δ*ATG*5-4: two *Atg5* knock-out mutant strains. Germination rate of under glucose-relief condition was tested **(C)**. AO, wild type strain; glu(–), fungistatic condition; glu(+), glucose-relief condition; **,*P* < 0.01.

In order to evaluate the role of autophagy in resisting soil fungistasis, the genes *Atg1* and *Atg5* were independently knocked out. Two Δ*ATG1* mutants and two Δ*ATG5* mutants were obtained (Supplementary Figure S2). The germination rates of wild-type and mutant strains were tested (Figure 6). Although the germination rate of mutant strains at 4 h decreased slightly, the germination rates of wild-type and mutant strains were almost comparable on water agar medium (Figure 6A), indicating that disruption of autophagy did not affect conidial germination significantly. In soil suspension, however, the conidial germination rate of the Δ*ATG1* and Δ*ATG5* mutants decreased significantly (Figure 6B), especially in soil suspension with a water/soil ratio of 1:1 or 1:2.5. The germination rate of Δ*ATG1* mutants under glucose-relief condition was also tested (Figure 6C). The result showed that 1 ml of 1% glucose (w/v) could not relieve the conidia germination of Δ*ATG1* mutants. Although the germination rate (about 40%) of wild-type strain under the glucose-relief condition is less than that in Figure 1, it is higher significantly than that of wild-type strain under fungistatic condition. The different relief effect of glucose may well be resulted from different batches of soil collected in different seasons. These results suggest that the autophagy pathway plays an important role in resisting soil fungistasis, and in glucose-mediated relief of soil fungistasis.

## Discussion

Previous studies indicate that fungistasis is caused by a combination of nutrient deficiency and the inhibition of fungistatic factors in soils (Lockwood, 1977; Garbeva et al., 2011). Soil fungistasis mainly inhibits fungal spore germination and the growth of fungal hypha. Regarding *A. oligospora* soil fungistasis, the most likely mechanism behind inhibition of conidia germination is the existence of fungistatic factors in soils, because *A. oligospora* conidia displays normal germination in deionized water without any nutrients. Moreover, our recent studies indicated that two inhibitory factors, namely ammonia and benzaldehyde, could inhibit *A. oligospora* conidial germination; and that the repression of protein synthesis may be responsible for conidia germination inhibition (Liu et al., 2018, 2019). In this study, the analysis of transcriptome data indicated that approximately 80 genes related to protein synthesis are upregulated in fungistatic and glucose-relieved conidia, including ribosome structure and biogenesis genes and RNA transport genes. The upregulation of these genes may be a response to soil fungistasis.

Garbeva et al. (2011) indicated that exogenous nutrients may enable energy-driven efflux pumps to help fungi to resist soil fungistasis by pumping intracellular toxins out of conidia. The energy-driven efflux pumps include ATP-binding cassette (ABC) transporter and major facilitator superfamily (MFS) transporter, which are responsible for multidrug resistance (Monk and Goffeau, 2008; Schouten et al., 2008). Our results showed that several genes involved in oxidative phosphorylation were upregulated after glucose addition. This suggested that a small portion of added glucose might provide energy through the activated oxidative phosphorylation pathway, although most glucose must be diffused to outside of dialysis bag. Besides, glucose also activated the transcription of 21 transporter genes (Supplementary Table S6), including two ABC transporter genes, three MFS transporter genes, and ion channel genes. It remains to be explored whether energy-driven efflux pumps help in the resistance of soil fungistasis.

The expression of genes involved in membrane trafficking, autophagy, and mitophagy was also activated in glucose-relieved conidia. Membrane trafficking is involved in endocytosis, exocytosis (Zheng et al., 2015), autophagy (Soreng et al., 2018), and vesicle formation and fusion (Jurgens, 2004), and is, therefore, vital for fungal growth and differentiation. In this study, conidial germination rates of autophagy pathway mutants Δ*ATG1* and Δ*ATG5* decreased significantly in soil suspension. This result confirmed the important role of autophagy in resisting soil fungistasis, and thus glucose activation of the autophagy pathway would promote the conidial germination of glucose-relieved conidia.

In addition, the transcription of 15 cytoskeleton genes was also activated by glucose (Supplementary Table S6). The actin cytoskeleton proteins play important roles in various fundamental cellular processes, including the maintenance of cell shape, polarity, cell division, cell migration, endocytosis, and vesicular trafficking (Mishra et al., 2014). The tubulin cytoskeleton proteins play important roles in mitosis and meiosis (Carminati and Stearns, 1998). Thus, cytoskeleton proteins are involved in both substance transport and the cell cycle.

Aside from cytoskeleton proteins, genes encoding chromosome and associated proteins, DNA repair and recombination proteins, and DNA replication proteins are also indispensable in the cell cycle (Lin et al., 2017). Here, we observed 42, 33, and 16 genes in these categories, respectively, that were upregulated in glucose-relieved conidia (Supplementary Table S6). And upregulated genes with twofold changes in comparison group AO-Re/AO-So were also enriched in meiosis, DNA replication, and repair (Figure 4). After protein and RNA synthesis, DNA replication is an important event during conidial germination (Ayala and Rodriguez-Del, 1988). Moreover, spore germination is a process in which non-dividing haploid spores re-enter the mitotic cell cycle and resume vegetative growth (Joseph et al., 2007). Thus, the upregulation of genes in the above mentioned categories should promote the cell cycle and conidial germination process in *A. oligospora*. These observations are consistent with other reports. For example, glucose can modify cell proliferation in maize during germination (Lara-Núñez et al., 2017). Glucose in plant tissues triggers cell division (Gibson, 2004; Wang and Ruan, 2013) and cell cycle-related markers can be regulated at different levels by glucose in plants (Riou et al., 2000; Hartig and Beck, 2006).

Based on these reported findings and our transcriptome data, it can be concluded that, in addition to being a carbon and energy source, glucose is also a signaling molecule that help to relieve soil fungistasis by activating many cellular processes, including autophagy, DNA replication and repair, RNA alternative splicing, and degradation pathways.

## Data Availability Statement

The datasets generated for this study can be found in the National Omics Data Encyclopedia (NODE): OEP000440.

## Author Contributions

TL carried out the bioinformatical analysis of our data and wrote the manuscript. YH prepared the conidia sample, extracted the total RNA, and analyzed the data. X-XC, XL, Y-HY, and M-LZ revised the manuscript. M-HM and K-QZ designed and supervised the study.

## Conflict of Interest

M-LZ was employed by the company Yunnan of China National Tobacco Corporation. The remaining authors declare that the research was conducted in the absence of any commercial or financial relationships that could be construed as a potential conflict of interest.

## Footnotes

1 www.biosino.org

2 http://bowtie-bio.sourceforge.net/index.shtml

3 https://www.biostat.wisc.edu/∼cdewey/

4 https://www.kegg.jp/kegg/mapper.html

5 https://en.wikipedia.org/wiki/Hypergeometric_distribution

## References

[B1] Abad P. Gouzy J. Aury J. M. Castagnone-Sereno P. Danchin E. G. Deleury E. (2008). Genome sequence of the metazoan plant-parasitic nematode *Meloidogyne incognita*. *Nat. Biotechnol.* 26 909–915. 10.1038/nbt.1482 18660804

[B2] Adams P. B. Papavizas G. C. (1969). Survival of root-infecting fungi in soil. X. Sensitivity of propagules of *Thielaviopsis basicola* to soil fungistasis in natural. *Phytopathology* 59 135–138.

[B3] Ann P. J. (1994). Survey of soils suppressive to three species of *Phytophthora* in Taiwan. *Soil Biol. Biochem.* 26 1239–1248. 10.1016/0038-0717(94)90149-x

[B4] Atanasova L. Le C. S. Gruber S. Coulpier F. Seidl-Seiboth V. Kubicek C. P. (2013). Comparative transcriptomics reveals different strategies of *Trichoderma* mycoparasitism. *BMC genomics* 14:121. 10.1186/1471-2164-14-121 23432824 PMC3599271

[B5] Audic S. Claverie J. M. (1997). The significance of digital gene expression profiles. *Genome Res.* 7 986–995. 10.1101/gr.7.10.986 9331369

[B6] Ayala S. Rodriguez-Del V. N. (1988). Molecular and cellular events during the germination of conidia of *Sporothrix schenckii*. *Mycopathologia* 101 113–120. 10.1007/bf00452896 3344031

[B7] Butt T. M. Jackson C. W. Magan N. (2001). *Fungi as Biocontrol agents Progress, Problems and Potential.* Wallingford, UK: CABI Publishing.

[B8] Carminati J. L. Stearns T. (1998). “Chapter 6: cytoskeletal dynamics in yeast,” in *Methods in Cell Biology*, eds Sullivan K. F. Kay S. A. (Cambridge, MA: Academic Press), 87–105. 10.1016/s0091-679x(08)61950-0 9891376

[B9] Cock P. J. Fields C. J. Goto N. Heuer M. L. Rice P. M. (2010). The sanger FASTQ file format for sequences with quality scores, and the Solexa/Illumina FASTQ variants. *Nucleic Acids. Res.* 38 1767–1771. 10.1093/nar/gkp1137 20015970 PMC2847217

[B10] Deng G. M. Yang Q. S. He W. D. Li C. Y. Yang J. Zuo C. W. (2015). Proteomic analysis of conidia germination in *Fusarium oxysporum* f. sp. cubense tropical race 4 reveals new targets in ergosterol biosynthesis pathway for controlling Fusarium wilt of banana. *Appl. Microbiol. Biotechnol.* 99 7189–7207. 10.1007/s00253-015-6768-x 26129952

[B11] Dobbs C. G. Hinson W. H. (1953). A widespread fungistasis in soils. *Nature* 172 197–199. 10.1038/172197a0 13087151

[B12] Emmatty D. A. Green R. J. Jr. (1967). The role of nutrients and pH in reversing fungistasis of conidia of *Trichoderma viride*. *Can. J. Microbiol.* 13 635–642. 10.1139/m67-084 6068131

[B13] Fang L. Z. Kun X. C. Song Z. C. Qin X. J. Qiu H. Y. Qun D. C. (2011). Fungistatic intensity of agricultural soil against fungal agents and phylogenetic analysis on the *Actinobacteria* involved. *Curr. Microbiol.* 62 1152–1159. 10.1007/s00284-010-9836-6 21161228

[B14] Garbeva P. Hol W. H. Termorshuizen A. J. Kowalchuk G. A. De B. W. (2011). Fungistasis and general soil biostasis – A new synthesis. *Soil Biol. Biochem.* 43 469–477. 10.1016/j.soilbio.2010.11.020

[B15] Gibson S. I. (2004). Sugar and phytohormone response pathways: navigating a signalling network. *J. Exp. Bot.* 55 253–264. 10.1093/jxb/erh048 14673024

[B16] Hartig K. Beck E. (2006). Crosstalk between auxin, cytokinins, and sugars in the plant cell cycle. *Plant Biol.* 8 389–396. 10.1055/s-2006-923797 16807832

[B17] Joseph S. D. Zenvirth D. Simchen G. Barkai N. (2007). Spore germination in *Saccharomyces cerevisiae*: global gene expression patterns and cell cycle landmarks. *Genome Biol.* 8:R241. 17999778 10.1186/gb-2007-8-11-r241 PMC2258198

[B18] Jurgens G. (2004). Membrane trafficking in plants. *Annu. Rev. Cell. Dev. Biol.* 20 481–504. 10.1146/annurev.cellbio.20.082503.103057 15473849

[B19] Kim D. Langmead B. Salzberg S. L. (2015). HISAT: a fast spliced aligner with low memory requirements. *Nat. Methods* 12 357–360. 10.1038/nmeth.3317 25751142 PMC4655817

[B20] Kong L. Zhang Y. Ye Z. Q. Liu X. Q. Zhao S. Q. Wei L. (2007). CPC: assess the protein-coding potential of transcripts using sequence features and support vector machine. *Nucleic Acids. Res.* 35 345–349. 17631615 10.1093/nar/gkm391 PMC1933232

[B21] Lai Y. Liu K. Zhang X. Zhang X. Li K. Wang N. (2014). Comparative genomics and transcriptomics analyses reveal divergent lifestyle features of nematode endoparasitic fungus *Hirsutella minnesotensis*. *Genome Biol. Evol.* 6 3077–3093. 10.1093/gbe/evu241 25359922 PMC4255773

[B22] Langmead B. Salzberg S. L. (2012). Fast gapped-read alignment with Bowtie 2. *Nat. Methods* 9 357–359. 10.1038/nmeth.1923 22388286 PMC3322381

[B23] Lara-Núñez A. García-Ayala B. B. Garza-Aguilar S. M. Flores-Sánchez J. Sánchez-Camargo V. A. Bravo-Alberto C. E. (2017). Glucose and sucrose differentially modify cell proliferation in maize during germination. *Plant Physiol. Biochem.* 113 20–31. 10.1016/j.plaphy.2017.01.018 28157579

[B24] Leng W. Liu T. Li R. Yang J. Wei C. Zhang W. (2008). Proteomic profile of dormant *Trichophyton Rubrum* conidia. *BMC Genomics*. 9:303. 10.1186/1471-2164-9-303 18578874 PMC2443143

[B25] Li B. Dewey C. N. (2011). RSEM: accurate transcript quantification from RNA-Seq data with or without a reference genome. *BMC Bioinf.* 12:323. 10.1186/1471-2105-12-323 21816040 PMC3163565

[B26] Lin A. B. McNeely S. C. Beckmann R. P. (2017). Achieving precision death with cell-cycle inhibitors that target DNA replication and repair. *Clin. Cancer. Res.* 23 3232–3240. 10.1158/1078-0432.CCR-16-0083 28331049

[B27] Liu F. (2019). *Research on the ROLES of Gα Subunits and Autophagy Genes in Resisting the Soil Fungistasis in Arthrobotrys Oligospora.* Kunming: Yunnan University.

[B28] Liu T. Tian D. W. Zou L. J. Liu F. Y. Can Q. Y. Yang J. K. (2018). Quantitative proteomics revealed partial fungistatic mechanism of ammonia against conidial germination of nematode-trapping fungus *Arthrobotrys oligospora* ATCC24927. *Int. J. Biochem. Cell Biol.* 98 104–112. 10.1016/j.biocel.2018.03.009 29544894

[B29] Liu T. Zou L. J. Tian D. W. Can Q. Y. Zhu M. L. Mo M. H. (2019). Proteomic changes in *Arthrobotrys oligospora* conidia in response to benzaldehyde-induced fungistatic stress. *J. Proteom.* 192 358–365. 10.1016/j.jprot.2018.09.016 30282050

[B30] Lockwood J. L. (1964). Soil Fungistasis. *Annu. Rev. Phytopathol.* 2 341–362.

[B31] Lockwood J. L. (1977). Fungistasis in soils. *Biol. Rev.* 52 1–43. 10.1111/j.1469-185x.1977.tb01344.x

[B32] Mishra M. Huang J. Balasubramanian M. K. (2014). The yeast actin cytoskeleton. *FEMS Microbiol. Rev.* 38 213–227. 10.1111/1574-6976.12064 24467403

[B33] Monk B. C. Goffeau A. (2008). Outwitting multidrug resistance to antifungals. *Science* 321 367–369. 10.1126/science.1159746 18635793

[B34] Oh Y. T. Ahn C. S. Kim J. G. Ro H. S. Lee C. W. Kim J. W. (2010). Proteomic analysis of early phase of conidia germination in *Aspergillus nidulans*. *Fungal Genet. Biol.* 47 246–253. 10.1016/j.fgb.2009.11.002 19919853

[B35] Papavizas G. C. Kovacs M. F. J. (1972). Stimulation of spore germination of *Thielaviopsis basicola* by fatty acids from rhizosphere soil. *Phytopathology* 62 688–694.

[B36] Pertea M. Pertea G. M. Antonescu C. M. Chang T. C. Mendell J. T. Salzberg S. L. (2015). StringTie enables improved reconstruction of a transcriptome from RNA-seq reads. *Nat. Biotechnol.* 33 290–295. 10.1038/nbt.3122 25690850 PMC4643835

[B37] Riou K. C. Menges M. Healy J. M. S. Murray J. A. H. (2000). Sugar control of the plant cell cycle: differential regulation of *Arabidopsis* D-type cyclin gene expression. *Mol. Cell. Biol.* 20 4513–4521. 10.1128/mcb.20.13.4513-4521.2000 10848578 PMC85832

[B38] Schouten A. Maksimova O. Cuesta A. Y. Van D. B. G. Raaijmakers J. M. (2008). Involvement of the ABC transporter BcAtrB and the laccase BcLCC2 in defence of *Botrytis cinerea* against the broad-spectrum antibiotic 2,4-diacetylphloroglucinol. *Environ. Microbiol.* 10 1145–1157. 10.1111/j.1462-2920.2007.01531.x 18218030

[B39] Shen S. Park J. W. Lu Z. X. Lin L. Henry M. D. Wu Y. N. (2014). rMATS: robust and flexible detection of differential alternative splicing from replicate RNA-Seq data. *Proc. Natl. Acad. Sci. U.S.A.* 111 E5593–E5601. 10.1073/pnas.1419161111 25480548 PMC4280593

[B40] Singh U. B. Sahu A. Sahu N. Singh R. K. Renu S. Singh D. P. (2013). Arthrobotrys oligospora-mediated biological control of diseases of tomato (*Lycopersicon esculentum* Mill.) caused by *Meloidogyne incognita* and *Rhizoctonia solani*. *J. Appl. Microbiol.* 114 196–208. 10.1111/jam.12009 22963133

[B41] Singh U. B. Sahu A. Singh R. K. Singh D. P. Meena K. K. Srivastava J. S. (2012). Evaluation of biocontrol potential of *Arthrobotrys oligospora* against *Meloidogyne graminicola* and *Rhizoctonia solani* in Rice (*Oryza sativa* L.). *Biol. Control* 60 262–270. 10.1016/j.biocontrol.2011.10.006

[B42] Soreng K. Neufeld T. P. Simonsen A. (2018). Membrane trafficking in autophagy. *Int. Rev. Cell. Mol. Biol.* 336 1–92. 10.1016/bs.ircmb.2017.07.001 29413888

[B43] Sun M. Liu X. Lin T. (1997). Fungistatic effect of soils on nematophagous fungi and their preparations. *Mycosystema* 16 149–154.

[B44] Tunlid A. Ahman J. Oliver R. P. (1999). Transformation of the nematode-trapping fungus *Arthrobotrys oligospora*. *FEMS Microbiol. Lett.* 173 111–116. 10.1016/s0378-1097(99)00059-2 10220888

[B45] Vlahakis A. Lopez M. N. Powers T. (2017). Stress-response transcription factors Msn2 and Msn4 couple TORC2-Ypk1 signaling and mitochondrial respiration to ATG8 gene expression and autophagy. *Autophagy* 13 1804–1812. 10.1080/15548627.2017.1356949 29198169 PMC5788474

[B46] Wang L. Ruan Y. L. (2013). Regulation of cell division and expansion by sugar and auxin signaling. *Front. Plant. Sci.* 4:163. 10.3389/fpls.2013.00163 23755057 PMC3667240

[B47] Zheng H. Zheng W. Wu C. Yang J. Xi Y. Xie Q. (2015). Rab GTPases are essential for membrane trafficking-dependent growth and pathogenicity in *Fusarium graminearum*. *Environ. Microbiol.* 17 4580–4599. 10.1111/1462-2920.12982 26177389

