# Supplementary material for: Comparative Transcriptomics Reveals Features and Possible Mechanisms of Glucose-Mediated Soil Fungistasis Relief in Arthrobotrys oligospora
Source: Front Microbiol. 2020 Jan 23;10:3143. doi: 10.3389/fmicb.2019.03143 (PMC6989558; doi:10.3389/fmicb.2019.03143)
Supplement: Supplementary file 1 [file Data_Sheet_1.docx]

1. The glucose concentration was assessed by blood glucose meter (Table S1)

Table S1 glucose concentration change during glucose-mediated relief of soil fungistasis. AO-Re: glucose-relieved conidia. AO-So: fungistatic conidia. Control: soil suspension with a mass ratio of 2.5/1.

| Sample | glucose concentration (mmol/L) | |
| --- | --- | --- |
|  | 0h | 24h |
| AO-Re | 14.8±0.2 | <1 |
| AO-So | <1 | <1 |
| Control | <1 | <1 |

2. Primers used for gene knock-out were listed Table S2.

Table S2 Primers used for knocking out gene *Atg1* and *Atg 5*

| Primer name | Primer sequence 5′→3′ | Description |
| --- | --- | --- |
| *AOL_s00076g70-*5f | CAGCAGCAACTTATTAGCCC | Amplify the 5′ flank of gene *AOL_s00076g70* |
| *AOL_s00076g70-*5r | AGTGGTTCTCCCTGCCTTC |  |
| *AOL_s00076g70-*5f | TGCCTACTTTCCCTCGTC | Amplify the 3′ flank of gene *AOL_s00076g70* |
| *AOL_s00076g70-*5r | TTCCCTCAAAGCCCATAC |  |
| AOL_s00076g234-5f | GCTTACAGCGAACCAATGAG | Amplify the 5′ flank of gene AOL_s00076g234 |
| AOL_s00076g234-5r | GGAGGAGAGATAAAGGGCAA |  |
| AOL_s00076g234-3f | TCGGTGGCTACGAGACTTA | Amplify the 3′ flank of gene AOL_s00076g234 |
| AOL_s00076g234-3r | GCAAGGGTTGTAGTTTATTAGAG |  |

3. The quality of RNA used for transcriptome sequencing was check (Table S3).

Table S3 Quality check of total RNA.

| Samples | Concentration （ng/μL） | Total RNA（ug） | OD260/280 | OD260/230 | RNA Integrity Number | 28S/18S |
| --- | --- | --- | --- | --- | --- | --- |
| AO-Ck（1） | 1188 | 55.84 | 2.21 | 2.4 | 8.8 | 1.9 |
| AO-Ck（2） | 1410 | 69.09 | 2.21 | 2.2 | 8.3 | 1.8 |
| AO-G24（1） | 528 | 16.9 | 2.1 | 2.24 | 7.5 | 1.5 |
| AO-G24（2） | 406 | 12.18 | 2.16 | 2.15 | 7.1 | 1.6 |
| AO-So（1） | 260 | 10.4 | 2.07 | 2.01 | 7.6 | 1.7 |
| AO-So（2） | 70 | 1.4 | 2.04 | 1.43 | 7.4 | 1.4 |
| AO-Re（3） | 228 | 7.75 | 2.09 | 1.72 | 7 | 1 |
| AO-Re（4） | 42 | 1.05 | 1.97 | 0.21 | 7.3 | 1 |

4. Reads filtering.

Table S4 Summary of sequencing reads after filtering

| Sample | Total Raw Reads(Mb) | Total Clean Reads(Mb) | Total Clean Bases(Gb) | Clean Reads Q20(%) | Clean Reads Q30(%) | Clean Reads Ratio(%) |
| --- | --- | --- | --- | --- | --- | --- |
| AO-Ck | 43.84 | 30.27 | 4.54 | 98.55 | 95.93 | 69.03 |
| AO-G24 | 47.09 | 29.54 | 4.43 | 98.53 | 95.84 | 62.73 |
| AO-Re | 35.63 | 30.21 | 4.53 | 98.19 | 94.73 | 84.8 |
| AO-So | 34.58 | 29.41 | 4.41 | 98.23 | 94.8 | 85.05 |

5. Genome mapping (Table S5).

Table S5 Summary of genome mapping

| Sample | Total Clean Reads | Total Mapping Ratio | Uniquely Mapping Ratio |
| --- | --- | --- | --- |
| AO-Ck | 30267082 | 80.44% | 80.16% |
| AO-G24 | 29541820 | 79.27% | 79.12% |
| AO-Re | 30213230 | 81.70% | 77.72% |
| AO-So | 29406102 | 84.15% | 83.40% |

Uniquely Mapping: Reads that map to only one location of reference

6. The distribution of base content and quality.
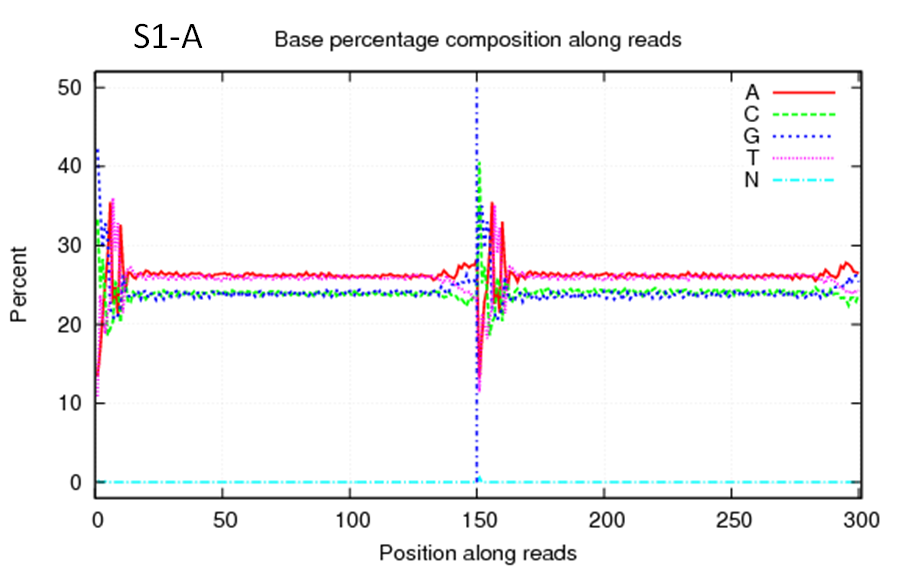


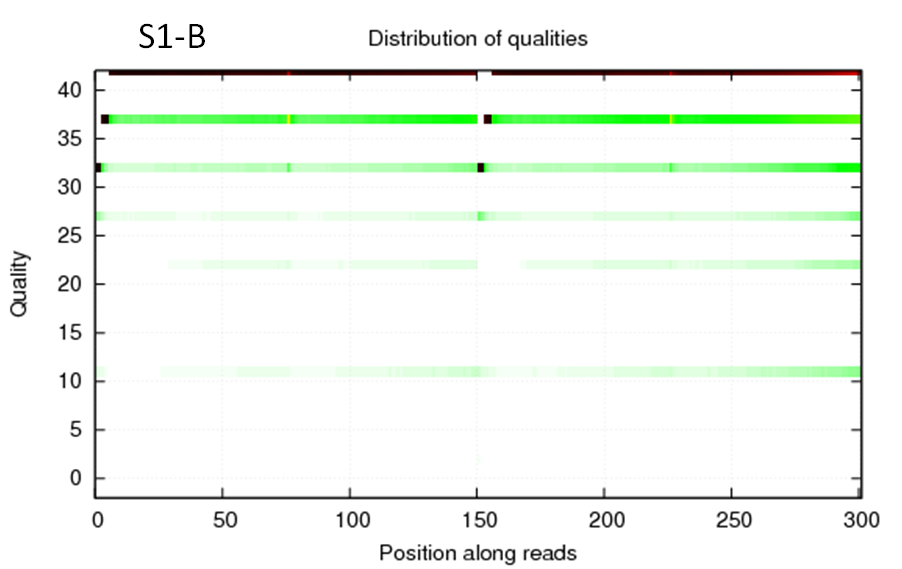


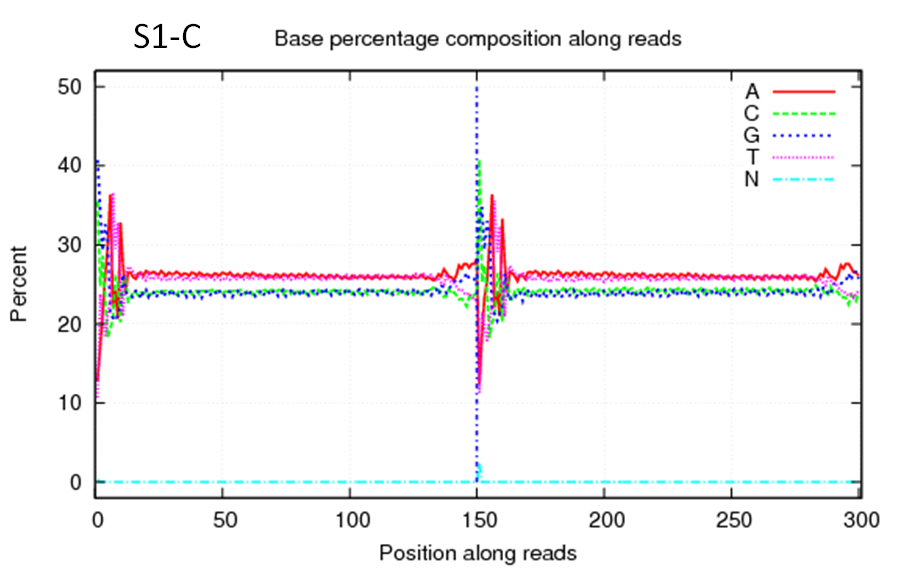


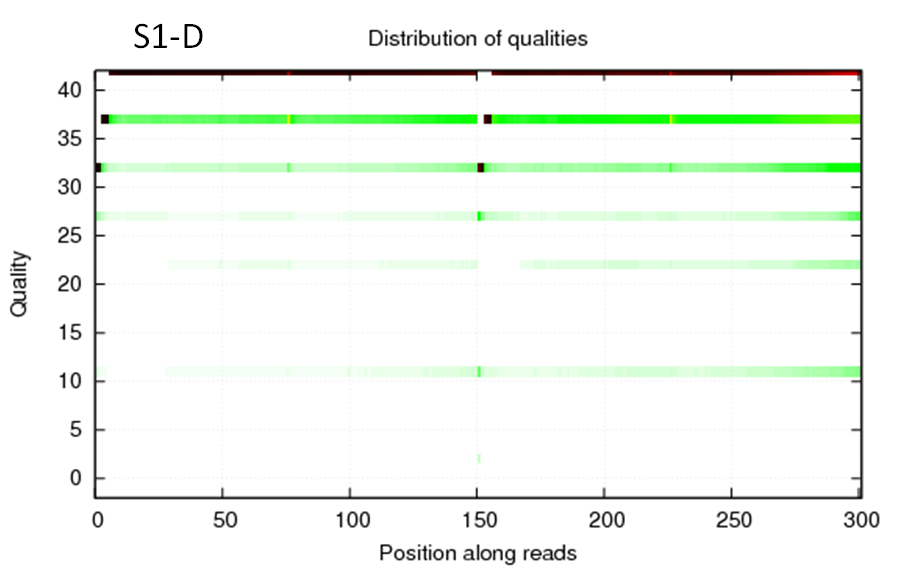


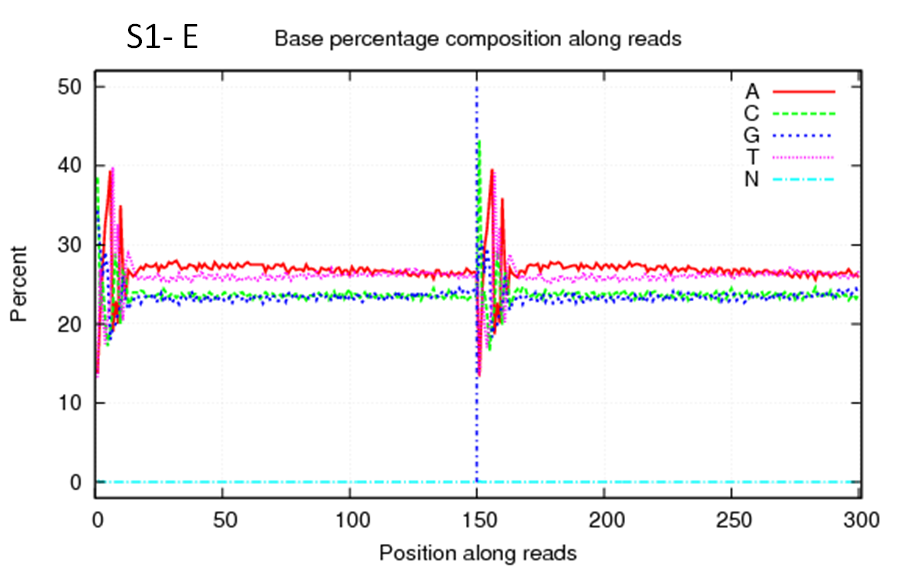


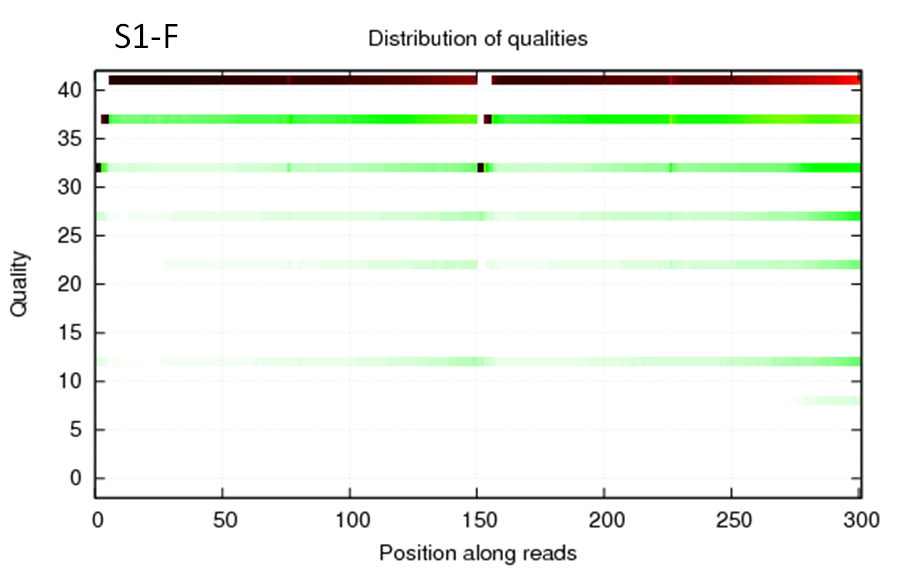


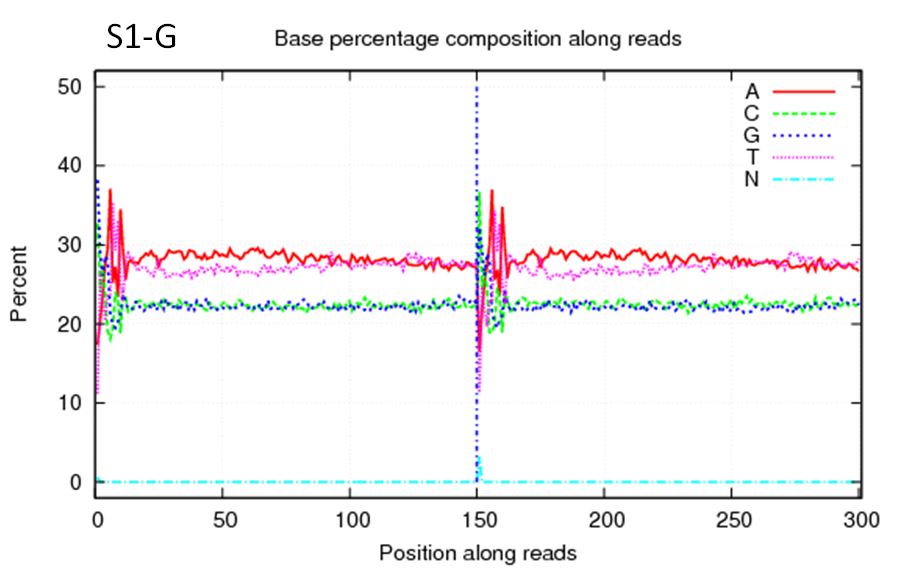


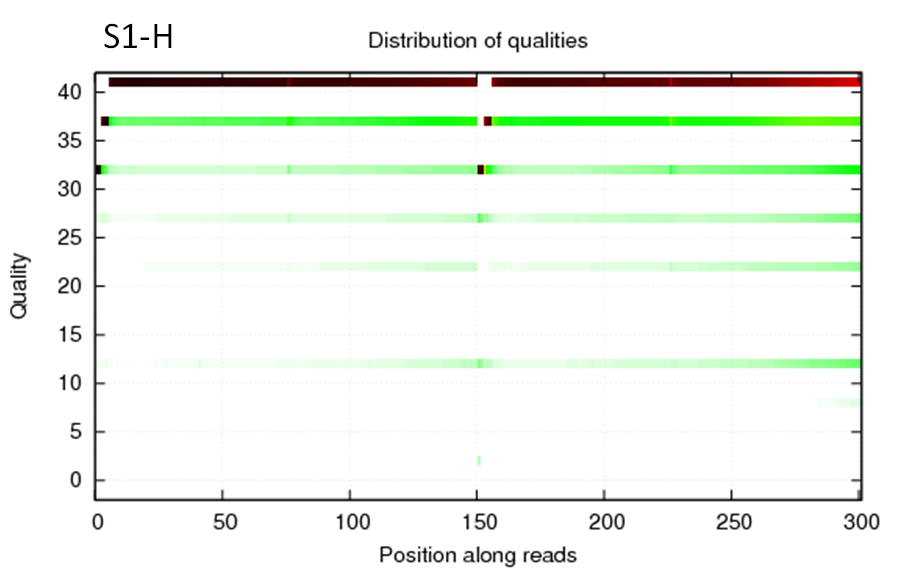


Figure S1| Distribution of base composition (S1-A, S1-C, S1-E, and S1-G) and base quality (S1-B, S1-D, S1-F, and S1-H) on clean reads. X axis represents base position along reads, Y axis represents base content percentage. AO-Ck: S1-A, S1-B; AO-G24: S1-C, S1-D; AO-So: S1-E, S1-F; AO-Re: S1-G, S1-H.

7. The hygromycin-resistant knock-out transformants were identified by PCR. The positive transformants were then subcultured for five transfers, and subcultured transformants were confirmed by PCR again (Figure S2).


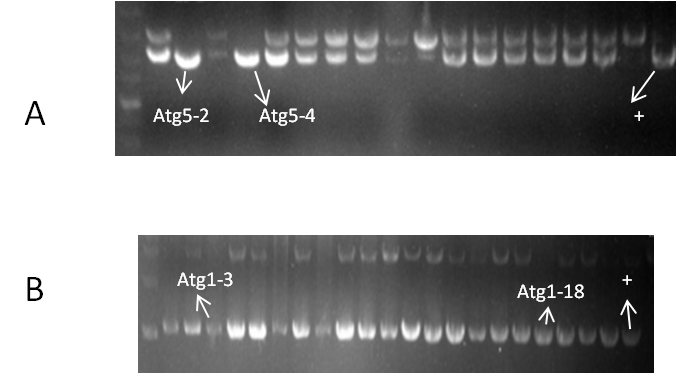


Figure S2| Knock-out mutant strains confirmation by PCR.A: two Atg5 gene knock-out mutants were confirmed. B: two Atg1 gene knock-out mutants were confirmed. +: plasmid was used as positive control.

8. KEGG Brites analysis of differentially expressed genes in fungistatic and glucose-relieved conidia

Table S6 KEGG Brites analysis of differentially expressed genes in fungistatic and glucose-relieved conidia

| KEGG Brites | AO-So | | AO-Re | |
| --- | --- | --- | --- | --- |
|  | up | down | up | down |
| ko01000 Enzymes | 140 | 132 | 237 | 73 |
| ko03036 Chromosome and associated proteins | 22 | 13 | 42 | 15 |
| ko04131 Membrane trafficking | 11 | 27 | 28 | 12 |
| ko03400 DNA repair and recombination proteins | 10 | 14 | 33 | 4 |
| ko04147 Exosome | 15 | 13 | 24 | 7 |
| ko02000 Transporters | 18 | 23 | 21 | 8 |
| ko03019 Messenger RNA biogenesis | 12 | 6 | 22 | 5 |
| ko03021 Transcription machinery | 7 | 7 | 21 | 7 |
| ko04121 Ubiquitin system | 5 | 9 | 16 | 9 |
| ko03029 Mitochondrial biogenesis | 14 | 14 | 16 | 7 |
| ko01002 Peptidases | 3 | 9 | 19 | 2 |
| ko03009 Ribosome biogenesis | 52 | 3 | 17 | 2 |
| ko03032 DNA replication proteins | 6 | 3 | 16 | 4 |
| ko03041 Spliceosome | 6 | 4 | 14 | 4 |
| ko04812 Cytoskeleton proteins | 3 | 5 | 15 | 2 |
| ko03000 Transcription factors | 6 | 6 | 6 | 12 |
| ko01009 Protein phosphatases/associated proteins | 1 | 7 | 10 | 7 |
| ko03110 Chaperones and folding catalysts | 4 | 3 | 12 | 2 |
| ko03016 Transfer RNA biogenesis | 13 | 6 | 13 | 1 |
| ko01001 Protein kinases | 3 | 7 | 8 | 5 |
| ko03051 Proteasome | 0 | 3 | 9 | 3 |
| ko01003 Glycosyltransferases | 4 | 5 | 8 | 3 |
| ko03011 Ribosome | 54 | 3 | 9 | 0 |
| ko03012 Translation factors | 6 | 3 | 5 | 2 |
| ko01007 Amino acid related enzymes | 9 | 3 | 4 | 1 |
